# Mobile Phone Use and Cognitive Impairment among Elderly Chinese: A National Cross-Sectional Survey Study

**DOI:** 10.3390/ijerph18115695

**Published:** 2021-05-26

**Authors:** Shige Qi, Yuying Sun, Peng Yin, Han Zhang, Zhihui Wang

**Affiliations:** 1 National Center for Chronic and Noncommunicable Disease Control and Prevention, Chinese Center for Disease Control and Prevention, Beijing 100050, China; qishige@ncncd.chinacdc.cn (S.Q.); yinpeng@ncncd.chinacdc.cn (P.Y.); zhanghan@ncncd.chinacdc.cn (H.Z.); 2 School of Public Health, The University of Hong Kong, Hong Kong, China; gyysun@hku.hk

**Keywords:** cognitive impairment, cognitive function, mobile phone, elderly, dementia, smartphone

## Abstract

The study aimed to investigate the relationship between mobile phone use and cognitive impairment using the data of the Prevention and Intervention on Neurodegenerative Disease for Elderly in China (PINDEC) survey. A total of 21,732 participants aged 60 years and above in China were recruited using a stratified, multi-stage cluster sampling method, providing information on demographics, lifestyle and health-related characteristics, mobile phone use, and cognitive impairment through face-to-face interviews by trained staff according to a standard protocol. All estimates of rates were weighted by sex, age, and living area (rural or urban) in the elderly Chinese population. The rate of mobile phone usage was 65.5% (14.3% for smartphone use). The prevalence of cognitive impairment in non-users of mobile phone, dumbphone users, and smartphone users were 17.8%, 5.0%, and 1.4%, respectively. The odds of having cognitive impairment in users of dumbphone and smartphone were lower than non-users after adjusting for demographics, lifestyle, and health-related factors (adjusted odds ratio (AOR), 0.39, 95% CI 0.35 to 0.45; *p* < 0.001; AOR, 0.16, 95% CI 0.11 to 0.25; *p* < 0.001, respectively). Smartphone use in Chinese elderly people was quite low. A strong correlation was found between mobile phone use and better cognitive function; yet longitudinal studies are warranted to explore the causal relationship. Future design of mobile phone-based interventions should consider the feasibility among those in need.

## 1. Introduction

China’s aging population is rising rapidly. The proportion of people aged 60 and over was 17.4% in 2020 [1], which is estimated to increase to 34.6% in 2050 [1]. This upward trend indicates that China is likely to face increased burden of age-related diseases, such as cognitive impairment. Mild cognitive impairment (MCI) is defined as cognitive decline greater than that expected for an individual’s age and education level but does not interfere notably with activities of daily life [2]. A systematic review in 2018 showed an 14.7% prevalence of MCI in China [3]. A recent national survey conducted in China from 2015 to 2018 reported that dementia affects 6% of adults aged 60 years and above, and MCI affects 15.5% [4]. A meta-analysis of 41 robust inception cohort studies summarized that less than half of individuals with MCI tend to progress to dementia in 3–10 years [5], and the annual progression rate is 5–15% [5,6]. A longitudinal study in Chinese elderly people from 2005 to 2014 found more than half of participants reported a decline of cognitive function [7]. Cognitive impairment and dementia lead to inconvenience of elderly people’s independent living and impose a heavy burden on families and society [8]. Exploring potentially modifiable risk factors is necessary for preventing the incidence of dementia or MCI. A most recent review and meta-analysis reported low social contact and depression were two of the risk factors of dementia [8]. A study in China showed similar risk factors of dementia and MCI, including rural residence, less education, unmarried, smoking, hypertension, hyperlipidaemia, diabetes, heart disease, and cerebrovascular disease [4]. However, most of these factors are less likely to be modified.

Nowadays, information and communication technology, such as mobile phones and computers, have been incorporated into people’s daily lives, reforming the way of communication and social connection. The 43rd Statistical Report on Internet Development in China showed that among the 829 million Internet users (817 million mobile phone users), by 2018, adults aged 60+ years accounted for 6.6% in all age groups [9]. However, the prevalence of mobile phone usage in Chinese elderly people was unknown. A 2016 survey in Xiamen in China showed 30% of adults aged 60+ years used smartphones, and more functions were positively associated with increased general and partial subdomain cognitive functions [10]. Another small-scale, cross-sectional survey in China showed association between the usage of mobile phone and cognitive function [11]. Since social contact and intellectual activities were both identified as protective factors of cognitive impairment [8,12], the mobile phone users may have better cognitive function due to minimized social isolation and enhanced cognitive skills.

Digital technology may be effective in improving elderly people’s quality of life and facilitating their social participation and involvement into communities, although there are concerns, such as personal security and privacy [13]. A large-scale study in China reported that both Internet access and mobile phone usage were associated with lower levels of depression [14]. Mobile phone apps have been designed to address the need for caregiving and self-care management in patients with dementia [15,16]. The main features of the apps were reminders, healthcare tips, and social network enhancement [16]. A 2021 systematic review identified a few studies evaluating the effectiveness of digital technologies used by people with dementia, although no inclusive findings could be provided about their effectiveness in improving participants’ self-management or social participation [17]. Since mobile-phone-based interventions have potential in improving quality of life, dementia care, and caregiving [15], it is necessary to understand mobile phone usage in the elderly group.

However, there is a lack of population-based study on the rate of mobile phone use in Chinese elderly people and its relationship with cognitive impairment independent of other risk factors. Therefore, we conducted this study to investigate the relationship between mobile phone use and cognitive impairment with the data of the Prevention and Intervention on Neurodegenerative Disease for Elderly in China (PINDEC).

## 2. Materials and Methods

### 2.1. Participants

The PINDEC study was initiated in 2015 aiming to explore the epidemiology of neurodegenerative diseases and associated risk factors among 24,117 adults aged 60 years and above in China [18]. There have been four waves of survey from 2015 to 2018. The baseline survey in 2015 included information about demographics, lifestyle, physical measurement, and biochemical tests. As least one wave of the follow-up surveys in 2016–2018 collected data on mortality, hospitalization, clinical assessments on Alzheimer’s disease and Parkinson’s disease, caregiver needs, depression, activities of daily living, and fall risk assessment, etc. Only the third wave in 2017 involved data of mobile phone use. In the present study, we utilized the baseline and the follow-up survey in 2017, including 22,009 participants (91.3%). Those records with missing data from any of the variables used in the present study were defined as incomplete data. After excluding 277 records with missing data, 21,732 participants were included.

Ethical approval was granted by the ethical review committee of the National Center for Chronic and Noncommunicable Disease Control and Prevention, Chinese Center for Disease Control and Prevention. All the participants provided written informed consent prior to the survey.

### 2.2. Sampling Method

The subjects in the PINDEC study were recruited using a stratified multi-stage cluster sampling method with six steps: (1) six provincial administrative units were selected according to the geographic location, population size, and level of economic development: Beijing, Shanghai, Hubei, Sichuan, Guangxi, and Yunnan; (2) one urban district and one rural county were randomly selected within each provincial administrative unit; (3) one subdistrict in urban districts or one township in rural counties was selected with probability proportional to population size; (4) four to eight neighborhood communities or administrative villages were selected through probability proportional to population size within each subdistrict or township; (5) 100 to 200 households with people aged 60 years and older from each neighborhood community or administrative village were randomly selected as study households; (6) all family members aged 60 years and older who registered as residents and lived in the households for more than one year prior to the survey were chosen as participants.

### 2.3. Data Collection

Face-to-face interview was conducted to collect data by trained staff according to a standard protocol, either in the participants’ households or in examination centers at local health stations within the participants’ residential areas. To ensure the validity and reliability of the data, interviewers and laboratory staff underwent intensive training followed by written and practical tests. Only qualified staff could conduct data collection on the field sites. All questionnaires were administered using a tablet with automatic skip pattern and logic check. All interviews were recorded, and 5% of the questionnaires were randomly selected for quality check.

### 2.4. Sociodemographic Characteristics, Lifestyle and Health-Related Variables

In the 2015 baseline survey, sociodemographic variables included age, sex, education level, marital status, living area, and occupation. Lifestyle variables included smoking status, alcohol drinking, tea drinking, physical exercise, living with family or alone, socializing with neighbors, and newspaper reading. Health-related variables involved body mass index (BMI), hypertension, diabetes, coronary heart disease (CHD), stroke, and hearing loss. These factors were collected for their possible association with cognitive function [3,4,8,12].

Body weight, height, and waist circumference were measured according to a standard protocol, and BMI was calculated. Based on the criteria of China Obesity Working Group [19], normal weight was defined as 18.5 ≤ BMI < 24.0, low weight as BMI < 18.5, overweight as 24.0 ≤ BMI < 28.0, and obesity as BMI ≥ 28.0. With an automated device, blood pressure was measured at the nondominant arm 3 times consecutively with a 1-min interval between the measurements in a seating position after 5 min of rest. The self-reported data of diagnosed CHD, stroke, and hearing loss were collected.

### 2.5. Mobile Phone Use

In the 2017 follow-up survey, participants were asked about mobile phone use through a question: “Are you currently using a mobile phone?”. The choices were “not using mobile phone”, “using dumbphone”, or “using smartphone”. The survey was administered by trained staff who would explain the functions of dumbphone and smartphone if the difference was unclear to the participants. Dumbphones typically provide voice calling and text messaging functions in addition to basic multimedia and Internet capabilities. Smartphones provide wider functions for multimedia and Internet. One additional question, “Why did you not use a smartphone?”, was answered by those using dumbphones, with three choices provided (“too expensive”/“don’t know how to use it”/“feels unnecessary”.). Three additional yes-or-no questions were collected from those using smartphones: “Do you access the Internet frequently?”, “Do you like to check health-related information online?”, and “Would you like to receive health and chronic disease management via mobile phone?”.

### 2.6. Cognitive Impairment

In the 2017 follow-up survey, cognitive function was assessed with a Chinese version of Ascertain Dementia 8 (AD8) first [20]. AD8 is a brief instrument to screen cognitive impairment and has good diagnostic accuracy, taking only 3-min for administration time, on average [21]. The sensitivity and specificity of AD8 were 0.72–0.91 and 0.67–0.78 [21]. It features eight questions, including memory in relation to date, memory in relation to appointments, daily thought processes, finances, repetitive conversations, learning ability, hobby/activity level, and domains of judgement. Participants with AD8 score ≥ 2 were then assessed with a Chinese version of Mini-Mental State Examination (MMSE). The sensitivity and specificity of identifying dementia were, respectively, 85.2% and 92.7% [22]. Cognitive impairment was defined as MMSE ≤ 17 for illiterate (0 year of education), ≤20 for primary school (1–6 years of education), and ≤24 for junior high school and above (≥7 years of education) [22,23].

### 2.7. Statistical Analysis

As the electronic data collection and management system was adopted, the logic of the questions was well controlled to reduce missing data. For data cleaning, the outliers, including the logical errors and those beyond the reasonable range, were analyzed and cleaned by checking the frequency tables and box-plots. Demographic characteristics of the categorical variables were described in percentages in the overall population and in subgroups. All estimates of rate were weighted by sex, age, and living area (rural or urban) distributions in people aged 60 years and above of the 2010 Chinese population. A multivariable logistic regression was used to examine the association of mobile phone use with cognitive impairment. Three models were built, including a crude model, a model adjusting for demographics, and a full model including all the demographics, lifestyle factors, and health-status-related variables. The adjusted odds ratios (AORs) were reported. Multicollinearity was measured by assessing the pairwise correlation coefficients between any pair of independent variables. The correlation coefficients between predictor variables of |*r*| > 0.7 were defined as the presence of multicollinearity [24]. All *p* values were 2-tailed and a *p* value < 0.05 was considered statistically significant. All statistical analyses were conducted using SAS version 9.4 (SAS Institute Inc., Cary, NC, USA).

## 3. Results

### 3.1. Mobile Phone Use

**Figure 1:**
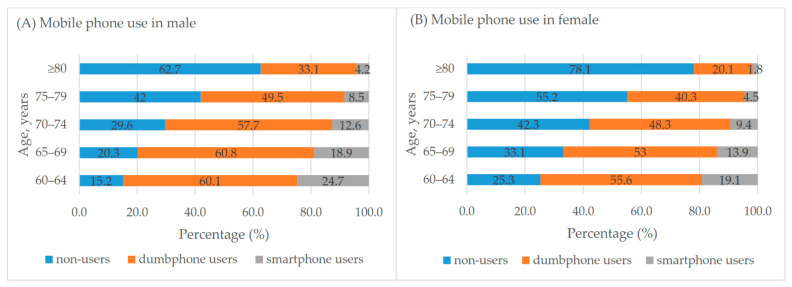
Mobile phone use by sex and age. (**A**) Mobile phone use in male; (**B**) Mobile phone use in female.

Overall, the weighted rate of mobile phone use in adults aged 60 and above was 65.5%, including 51.2% dumbphone users and 14.3% smartphone users. Table 1 shows mobile phone use in different populations. Sex, age, living area, marital status, education, occupation, and residing status were all associated with mobile phone use (all *p* < 0.001). Figure 1 shows mobile phone use by sex and age. Mobile phone use in both males and females decreased with age. In all age groups, males had higher mobile phone use than females (all *p* < 0.001).

Among those smartphone users, 50.8% had access to the Internet, 51.7% liked to check health-related information, and 58.6% were willing to receive health and chronic disease management via their smartphones. Those dumbphone users did not select smartphones due to the reasons of “don’t know how to use it” (85.9%), “feels unnecessary” (12.3%) or “too expensive” (1.8%).

### 3.2. Mobile Phone Use and Cognitive Impairment

**Figure 2:**
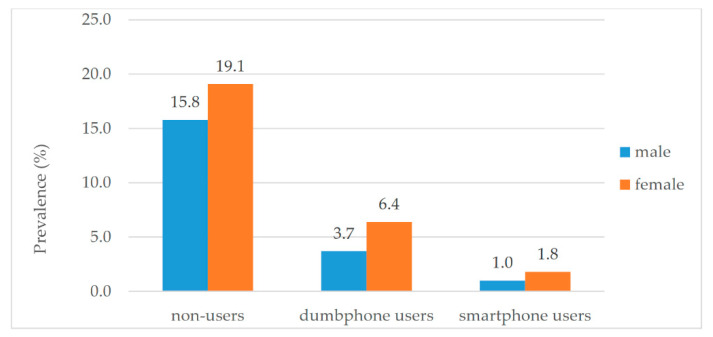
Prevalence of cognitive impairment in different mobile phone users.

The overall weighted prevalence of cognitive impairment in Chinese elderly people aged 60 or above was estimated at 8.9% (95% CI 8.5–9.3). Females had a higher prevalence of cognitive impairment (11.1%, 95% CI 10.5–11.7) than males (6.6%, 95% CI 6.1–7.1). Non-users of mobile phones, dumbphone users and smartphone users had significantly different weighted prevalence of cognitive impairment, which were 17.8%, 5.0%, and 1.4%, respectively (*p* < 0.001). Figure 2 shows the prevalence of cognitive impairment in males and females with different statuses of mobile phone use. Among the 14,223 mobile phone users, 95.8% did not have cognitive impairment.

Table 2 shows the three models studying the association between mobile phone use and cognitive impairment. No significant multicollinearity was found between the independent variables (all |*r*| < 0.2). All models showed strong associations between either dumbphone use or smartphone use and less cognitive impairment (better cognitive function). The crude model showed the odds of having cognitive impairment in users of dumbphone and smartphone were 0.24 (95% CI 0.22 to 0.27; *p* < 0.001) and 0.06 (95% CI 0.04 to 0.10; *p* < 0.001), respectively. The full model showed the odds of having cognitive impairment in users of dumbphone and smartphone were 0.39 (95% CI 0.35 to 0.45; *p* < 0.001) and 0.16 (95% CI 0.11 to 0.25; *p* < 0.001), respectively. The included demographic factors were sex, age, education, marital status, and occupation. Regarding the modifiable lifestyle factors, regular tea drinking, regular physical exercise, socializing with neighbors, and reading newspapers were all protective factors. Stroke history and hearing loss were correlated with higher levels of cognitive impairment (AOR, 2.56; 95% CI 2.08 to 3.14; *p* < 0.001; AOR, 1.61; 95% CI 1.28 to 2.01; *p* < 0.001, respectively). We found no significant association between cognitive impairment and living area, smoking, alcohol drinking, living with family, BMI, hypertension, CHD, or diabetes.

## 4. Discussion

In this nationwide study, we found a 65.5% overall rate of mobile phone use and 14.3% rate of smartphone use in Chinese elderly people aged 60 and above. Males, people aged 60–64, those living in urban areas, those non-widowed, those with higher levels of education, non-manual workers, and people living with families had higher rates of smartphone use (15.1–32.8%). The overall prevalence of cognitive impairment was 8.9%. Non-users of mobile phones, dumbphone users, and smartphone users had significantly different rates of cognitive impairment, which were 17.8%, 5.0%, and 1.4%, respectively. The availability of devices in elderly people should be considered when designing smartphone-based intervention programs.

Cognitive training or lifelong learning could prevent the cognitive decline [25]. A 2020 systematic review suggested information and communication-technology-based products should be offered to people with memory problems to support their activity and participation in everyday life [26]. In the present study, dumbphone users and smartphone users, respectively, had 61% and 84% lower risk of having cognitive impairment than those who were not using mobile phones. The mechanism of such lower risk may be due to both improved social communication and cognitive training. As dumbphones only include simple functions such as voice calling and text messaging, the effect on cognitive training might be very limited. Our study showed the majority of dumbphone users did not choose smartphones due to “don’t know how to use it”. This suggests the need for learning how to use smartphones in those dumbphone users. Older people may have hesitations to use more advanced functions, as they tend to rely on mobile phones for a sense of safety rather than communication [27]. We found more than half of smartphone users had access to the Internet or acquired health-related information via their phones. However, the present study did not show causal relationship between mobile phone use and better cognitive function. The decline in cognitive function might have caused the decreased smartphone use [28]. More ageing-friendly elements should be adopted when designing mobile phones for the elderly, especially for those who already have experienced cognitive decline [27].

Although mobile phones have potential benefits for cognitive performance, it is worth mentioning their possible negative health impact. The electromagnetic fields produced by mobile phones are classified by the International Agency for Research on Cancer as possibly carcinogenic to humans [29]. However, no short-term, negative effect of electromagnetic fields produced by mobile phones on cognitive function was found [30]. A four-year cohort study in Singapore among Chinese older people found no significant deleterious effect of digital mobile phone use on cognitive function [31]. The long-term effects are to be assessed by more studies.

For the other confounding factors included in the full model, we found both consistency and discrepancy between our study and the previous studies. Female, those of older age, people with lower education, those widowed, farmers, those who suffered hearing loss, and those who had stroke history were linked with an increased risk for cognitive impairment, while regular physical exercise, socializing with neighbors, and reading newspapers were all protective factors. The findings were consistent with the conclusions made in other studies [3,4,8,12]. Nevertheless, we found no significant associations between cognitive impairment and living area, smoking, alcohol drinking, living with family, BMI, hypertension, or coronary heart disease. Previous studies have shown living in rural areas, smoking, excessive alcohol consumption, obesity, diabetes, and hypertension were associated with higher levels of cognitive impairment [3,4,8]. The reasons for these different findings might be due to the adjustments made and different risk factors included in the model. Furthermore, the measurements lacked the information about the behavioral frequency and intensity. More robust measurements are needed in future studies. Genetic factors, such as mutation carriers (amyloid precursor protein, presenilin, and asynuclein) and apolipoprotein E (APOE) ε4 carriers, may be useful in determining the etiology of cognitive impairment [32,33]. Vitamin B12 and D deficiencies were also associated with increased risk of cognitive impairment [34,35]. Yet, these factors were not included in the present study, which might also play important role in cognitive decline.

Our study had several limitations. First, it was cross-sectional, and the association between mobile phone use and better cognitive function was not causal. In fact, it is possible that those elderly people with cognitive impairment were less motivated to interact with their social contacts via smartphones. Secondly, demographics and lifestyle factors were collected at baseline survey; some variables such as smoking, drinking, physical activity and tea drinking, socializing with neighbors, and reading newspapers might have been modified during the two-year follow-up. Third, the collected information on mobile phone use was not enough to determine its mechanism on affecting cognitive function. Lastly, the study is limited to China. The results are likely to differ in other countries and cultures due to the varied behavioral patterns of mobile phone use and environmental factors.

## 5. Conclusions

Mobile phone use is associated with better cognitive function in Chinese elderly people. With more robust measurements on the behavioral frequency and intensity, the causal relationship and mechanism should be explored by longitudinal studies. Future longitudinal studies should cover frequency and duration of mobile phone use, the function used, and in-depth information about the behavioral pattern. The low rate of mobile phone use, especially smartphone use, in Chinese elderly people should be taken into account in future research on prevention and intervention of cognitive impairment.

## Figures and Tables

**Table 1:** Mobile phone use in different populations.

Characteristics	n (%) ^1^	Mobile Phone Use			
		None n (%) ^1^	Dumbphone n (%) ^1^	Smartphone n (%) ^1^	*p* Values
Sex					
Male	10,651 (49.0)	2919 (27.4)	5936 (55.7)	1796 (16.9)	<0.001
Female	11,081 (51.0)	4591 (41.4)	5182 (46.8)	1309 (11.8)	
Age groups, years					
60–64	7179 (33.0)	1449 (20.2)	4155 (57.9)	1575 (21.9)	<0.001
65–69	5031 (23.2)	1340 (26.6)	2864 (56.9)	828 (16.5)	
70–74	4035 (18.6)	1452 (36.0)	2139 (53.0)	445 (11.0)	
75–79	2919 (13.4)	1429 (49.0)	1304 (44.6)	186 (6.4)	
≥80	2568 (11.8)	1840 (71.6)	657 (25.6)	72 (2.8)	
Living area					
Urban	9580 (44.1)	2617 (27.4)	4669 (48.7)	2294 (23.9)	<0.001
Rural	12,152 (55.9)	4892 (40.3)	6448 (53.0)	811(6.7)	
Marital status					
Widowed	4780 (22.0)	2307 (48.2)	2154 (45.1)	320 (6.7)	<0.001
Non-widowed	16,952 (78.0)	5202 (30.7)	8964 (52.9)	2785 (16.4)	
Education, years					
0	8808 (40.5)	4481 (50.8)	4005 (45.5)	322 (3.7)	<0.001
1–6	6898 (31.8)	1971 (28.6)	4118 (59.7)	809 (11.7)	
≥7	6026 (27.7)	1057 (17.6)	2994 (49.6)	1974 (32.8)	
Occupation					
Non-manual ^2^	3425 (15.8)	676 (19.7)	1662 (48.5)	1087 (31.8)	<0.001
Worker	4439 (20.4)	1057 (23.8)	2280 (51.4)	1102 (24.8)	
Farmer	13,868 (63.8)	5777 (41.7)	7176 (51.7)	916 (6.6)	
Residing status					
Living alone	2465 (11.3)	1021 (41.4)	1248 (50.7)	196 (7.9)	<0.001
Living with family	19,267 (88.7)	6489 (33.7)	9870 (51.2)	2909 (15.1)	
Total	21,732 (100.0)	7510 (34.5)	11,118 (51.2)	3105 (14.3)	

^1^ The percentages were weighted by sex, age, and living area (rural or urban) distributions in people aged 60 years and above of the 2010 Chinese population. ^2^ Non-manual worker includes teachers, researchers, doctors, office workers, and other occupations apart from farmer and worker.

**Table 2:** Logistic regression analysis of cognitive impairment in people with different statuses of mobile phone use.

Models	Variables	Reference Groups	OR/AOR (95% CI)
Crude	Dumbphone users	No mobile phone use	0.24 (0.22, 0.27) ***
	Smartphone users		0.06 (0.04, 0.10) ***
Adjusted for demographics	Dumbphone users	No mobile phone use	0.37 (0.33, 0.42) ***
	Smartphone users		0.14 (0.10, 0.22) ***
Full model	Dumbphone users	No mobile phone use	0.39 (0.35, 0.45) ***
	Smartphone users		0.16 (0.11, 0.25) ***
	Sex (female)	Sex (male)	1.24 (1.05, 1.46) *
	Aged 65–69 years	Aged 60–64 years	1.11 (0.92, 1.35)
	Aged 70–74 years		1.37 (1.13, 1.67) **
	Aged 75–79 years		2.00 (1.63, 2.43) ***
	Aged ≥ 80 years		3.43 (2.80, 4.20) ***
	Living in rural area	Living in urban area	1.03 (0.89, 1.19)
	Non-widowed	Widowed	0.80 (0.69, 0.92) **
	Education: 1–6 years	Education: 0 year	0.77 (0.67, 0.89) ***
	Education: ≥7 years		1.00 (0.79, 1.24)
	Worker	Non-manual worker	0.91 (0.70, 1.18)
	Farmer		1.62 (1.24, 2.12) ***
	Current smoker	Non-smoker	0.98 (0.81, 1.19)
	Former smoker		1.08 (0.87, 1.19)
	Alcohol drinking	No alcohol drinking	1.02 (0.88, 1.19)
	Regular tea drinking	No regular tea drinking	0.91 (0.78, 1.06) *
	Regular exercise	No regular exercise	0.83 (0.72, 0.95) **
	Living with family	Living alone	1.15 (0.96, 1.37)
	Socialize with neighbors occasionally	Almost never	0.58 (0.44, 0.77) ***
	Socialize with neighbors daily		0.51 (0.45, 0.59) ***
	Read newspapers occasionally	Almost never	0.70 (0.56, 0.88) **
	Read newspapers daily		0.62 (0.46, 0.84) ***
	BMI < 18.5	18.5 ≤ BMI < 24.0	1.08 (0.91, 1.30)
	24.0 ≤ BMI < 28.0		1.03 (0.90, 1.17)
	BMI ≥ 28.0		1.07 (0.88, 1.31)
	Hypertension	No hypertension	1.04 (0.93, 1.17)
	CHD	No CHD	0.99 (0.81, 1.20)
	Diabetes	No diabetes	1.00 (0.87, 1.16)
	Stroke	No stroke	2.56 (2.08, 3.14) ***
	Hearing loss	No hearing loss	1.61 (1.28, 2.01) ***

* *p*  <  0.05; ** *p*  <  0.01; *** *p*  <  0.001. OR, odds ratio; AOR, adjusted odds ratio; BMI, body mass index; CHD, coronary heart disease.

## Data Availability

The data presented in this study are available upon reasonable request from the corresponding author and approval of the funder.
